# Tuning Physical Properties of GelMA Hydrogels through Microarchitecture for Engineering Osteoid Tissue

**DOI:** 10.1021/acs.biomac.3c00909

**Published:** 2023-12-16

**Authors:** Ewa Walejewska, Ferry P. W. Melchels, Alessia Paradiso, Andrew McCormack, Karol Szlazak, Alicja Olszewska, Michal Srebrzynski, Wojciech Swieszkowski

**Affiliations:** † Faculty of Materials Science and Engineering, Warsaw University of Technology, Woloska 141, Warsaw 02-507, Poland; ‡ Centre for Advanced Materials and Technologies CEZAMAT, Warsaw University of Technology, Poleczki 19, Warsaw 02-822, Poland; § Institute of Biological Chemistry, Biophysics and Bioengineering, Heriot-Watt University, Edinburgh EH14 4AS, Scotland; ∥ Future Industries Institute, University of South Australia, Adelaide, South Australia 5095, Australia; ⊥ Department of Transplantology and Central Tissue Bank, Medical University of Warsaw, Chalubinskiego 5, Warsaw 02-004, Poland; # National Centre for Tissue and Cell Banking, Chalubinskiego 5, Warsaw 02-004, Poland

## Abstract

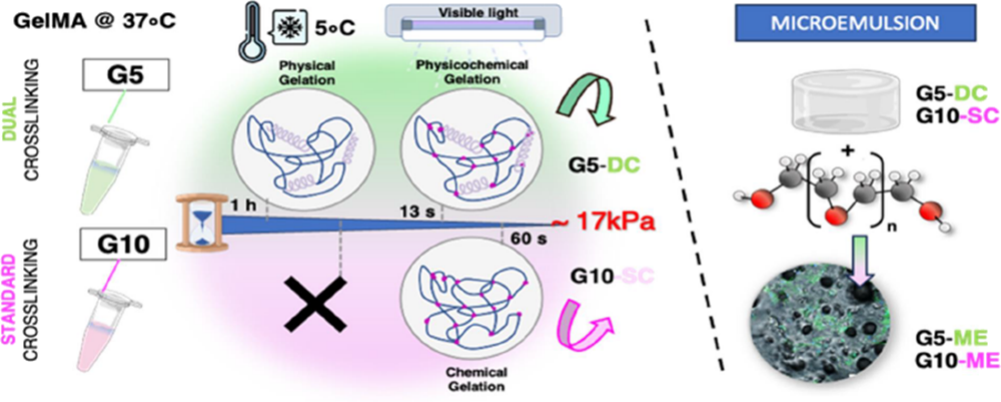

Gelatin methacryloyl (GelMA) hydrogels have gained significant attention due to their biocompatibility and tunable properties. Here, a new approach to engineer GelMA-based matrices to mimic the osteoid matrix is provided. Two cross-linking methods were employed to mimic the tissue stiffness: standard cross-linking (SC) based on visible light exposure (VL) and dual cross-linking (DC) involving physical gelation, followed by VL. It was demonstrated that by reducing the GelMA concentration from 10% (G10) to 5% (G5), the dual-cross-linked G5 achieved a compressive modulus of ∼17 kPa and showed the ability to support bone formation, as evidenced by alkaline phosphatase detection over 3 weeks of incubation in osteogenic medium. Moreover, incorporating poly(ethylene) oxide (PEO) into the G5 and G10 samples was found to hinder the fabrication of highly porous hydrogels, leading to compromised cell survival and reduced osteogenic differentiation, as a consequence of incomplete PEO removal.

## 1 Introduction

Bone is a unique environment that might be described as a composite material consisting of various types and levels of tissue organization. Its complexity is imposed on its ability to self-renew (bone marrow presence) in bone remodeling (BR), during which tightly related events and cell signaling pathways are distinguished.^1^ BR provides the microenvironment that links its two major stages—bone resorption and formation^2,3^ The knowledge of this mutual regulation and the approaches undertaken to recreate it remains a hot topic in regenerative medicine and tissue engineering.

In this frame, bone tissue engineering (BTE) aims to develop novel strategies for the regeneration of bone tissue using the combination of (bio)materials science, engineering strategies, and cell biology^4,5^ Despite the availability of autologous bone grafts and allografts, which could be transplanted on the side of a bone defect, there are drawbacks related to their limited accessibility, disease transmission, or/and inflammation possibility, which has compelled researchers to seek new alternatives to bone substitutes.^6^

BTE constructs are widely used in literature and focus on different fabrication approaches, material selection, and surface modification to meet bone structure requirements.^7,8^ Polyester-based materials are most frequently reported and combined with calcium particles to serve as a platform to induce cell attachment, proliferation, and permit maturation of the cell-laden structure into a healthy bone tissue.^8,9^

Although polyesters such as polycaprolactone and polyglycolide have many outstanding advantages such as low toxicity and processability, they have several limitations, for example, the inability to encapsulate cells, prolonged degradation, and high stiffness that might not fully recapitulate BR conditions.^10^

As previously mentioned, BR is a complex process, and in vitro-induced osteogenesis does not solely pertain to the immediate formation of mineralized tissue.^2^ Different types of cells are recruited during BR, where old or damaged bone tissue needs to be resorbed by osteoclasts prior to osteogenesis. As the bone formation process proceeds, it can be divided into two distinct phases: (i) osteoid (preosseous matrix) secretion by osteoblasts; and (ii) its mineralization to obtain calcified bone tissue.^3^ Osteoid, a complex interwoven network consisting mainly of collagen type I and bone matrix proteins, is a foundation for bone creation.^11^ Thus, its presence should not be neglected in the BTE approach and may be considered essential to answering the question “Is an exceedingly soft starting material required to achieve a calcified bone tissue in vitro?”.

To this aim, hydrogels have been recently used to construct templates guiding bone regeneration by promoting the differentiation of bone cells^12−14^ Although their stiffness is insufficient for obtaining mineralized and “ready-to-use” bone tissue, they might provide an ideal, easily tunable platform for osteogenic differentiation and osteoblasts’ preosseus matrix secretion^15,16^ Stiffness is a critical parameter in hydrogel-based BTE applications. In fact, it can dramatically influence cell behavior and tissue remodeling. By tailoring the mechanical properties of tissue-engineered constructs, hydrogels can mimic native tissues, parallelly regulating cell adhesion, proliferation, and differentiation. Indeed, the recreation of diverse tissue types can be achieved by regulating the material stiffness and in turn dictating the cell response, thus, making it a pivotal factor for BTE. Besides, hydrogels can also enhance a physiological-like cell response as cells are highly sensitive to their microenvironment.^17^

For this reason, another critical factor for successful BTE is the design and optimization of the hydrogel’s microarchitecture^18,19^ which considers the material structure, including pore size, interconnectivity, and pore geometry of the final construct.^20^ Among others, microarchitecture plays a pivotal role in determining cell behavior, nutrient diffusion, waste removal, and the formation of functional tissue constructs.^19^ Hence, the ability to precisely control and modify the microarchitecture of GelMA hydrogels is of paramount importance. Gelatin methacryloyl (GelMA) is one of the most currently used versatile hydrogels derived from gelatin^21−23^ Gelatin is a denatured form of collagen that is a significant component of the ECM in various tissues, including the osteoid matrix. Due to the modification of gelatin with methacryloyl groups, the use of GelMA enables control over the gelation kinetics and mechanical properties.^24^ Moreover, GelMA possesses several desirable properties, including biocompatibility, biodegradability, and the ability to be processed into three-dimensional (3D) structures.^25^

Several strategies have been proposed to manipulate the microarchitecture of GelMA hydrogels, including the coordination of gelation and chemical cross-linking. In a study conducted by Young et al., the effect of the dual-cross-linking approach on the mechanical properties of GelMA hydrogels was investigated.^26^ These findings revealed a significant increase in the storage modulus (*G*′), that is 8-fold higher for the hydrogels fabricated using this dual approach than those cross-linked solely through photoinitiated cross-linking. Moreover, similar to the work by Chansoria et al., thermal gelation followed by photo-cross-linking improved cell elongation and proliferation while maintaining the sample shape fidelity after fabrication.^27^

Another versatile approach for fabricating porous scaffolds with easily adjustable pore sizes is the emulsion methodology, which can be described as mixing two immiscible fluids, where one fluid forms droplets within the other.^28^ Among others, Pluronic F127, a block copolymer of poly(ethylene oxide) and poly(propylene oxide), remains the most popular option in tissue engineering and in combination with other polymers for the fabrication of sacrificial layers and porous structures^29,30^ The polymer solution is a liquid at room temperature (<25 °C), but gels rapidly at body temperature (37 °C). Although the polymer is not degraded by the body, the gels dissolve slowly and the polymer is eventually cleared^31,32^ However, due to the limitation of the micelle sizes, this approach has been challenging to fabricate constructs with large pore size.^33^ Thus, according to Ying et al. the emulsion of GelMA with poly(ethylene) oxide (PEO) addition might be an alternative for obtaining structures with hierarchical pore structures and controlled pore size^28,33,34^

Here, we aimed to optimize the microarchitecture of GelMA hydrogels to create a platform resembling an osteoid tissue. Two cross-linking methods were proposed: the standard cross-linking involving visible light exposure (VL) and a dual approach (DC) that combined physical gelation and subsequent VL fixation. GelMA concentrations and exposure times were tailored to result in hydrogels with the same stiffness but different microarchitectures.

To our knowledge, this is the first systematic study combining dual-cross-linking and porogen addition to obtain osteoid-like constructs. Furthermore, previous approaches that aimed at targeting the osteoid microarchitectures and mimicking BR-like conditions, such as gradual material degradation, are limited in the literature. In this study, we developed a novel approach for engineering osteoid-like scaffolds by replicating the properties of the osteoid tissue matrix. To this aim, two different cross-linking methods (i.e., SC and DC) were used. It was demonstrated that both achieved a compressive modulus similar to the native osteoid matrix, with G5 DC requiring only half the GelMA concentration, validating the potential of the synergistic effect of the two cross-linking modalities. The G5-DC formulation exhibited the ability to support bone formation during 3 weeks of in vitro incubation. The addition of PEO porogen limited the creation of highly porous hydrogels, thus impairing the osteogenic differentiation due to incomplete PEO extraction.

## 2 Experimental Section

### 2.1 GelMA Synthesis

GelMA was synthesized according to the previously established protocol with minor modifications.^35^ In brief, gelatin (porcine skin, type A, 300 g Bloom, Sigma-Aldrich) was reacted with methacrylic anhydride (Sigma-Aldrich) for 1 h at 50 °C. The methacrylic anhydride was added dropwise at 0.6 g of anhydride per gram gelatin, to a 10% (w/v) gelatin in PBS solution, under constant stirring. Prior to the addition of anhydride, the pH of the gelatin solution was adjusted to pH 8, with the addition of a 5 M NaOH solution. Following the reaction, excess anhydride was removed via centrifugation, decanting, and dialysis (cellulose membrane, cutoff 12 kDa, Sigma-Aldrich) against deionized water. The GelMA macromonomer solution was then neutralized, followed by storage at −20 °C for 48 h before freeze-drying and subsequent lyophilization. The as-prepared prepolymer was maintained at −20 °C until further use.

### 2.2 Quantification of Substitution of GelMA

The degree of functionalization (DoF) of GelMA macromonomers was quantified using proton-nuclear magnetic resonance spectroscopy (^1^H NMR, Bruker AVIII 300 MHz). Briefly, either unfunctionalized gelatin or the corresponding GelMA macromonomer was dissolved in deuterium oxide (D_2_O) at 37 °C, and a sample of 0.8 mL of either solution was used for the ^1^H NMR experiments. The DoF was then calculated from eq 1.
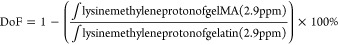
1

Prior to interpretation of the NMR spectra, gelatin and GelMA were normalized with respect to the phenylalanine signal at 7 ppm.

### 2.3 Fabrication of GelMA Hydrogel Samples

#### 2.3.1 Standard Cross-Linking and Dual-Cross-Linking Methods

The hydrogel prepolymer solution was prepared by soaking GelMA in PBS (5 and 10% (w/v)) at 4 °C overnight. Prior to this, in-house synthesized lithium phenyl-2,4,5-trimethylbenzoylphosphinate (LAP) was dissolved in PBS at a concentration of 0.1% (w/v). Subsequently, the GelMA solution was heated up to 40 °C, left stirring for 10 min, transferred to Teflon casting molds, and exposed to visible light (VL) for 10, 13, 30, and 60 s (GelMA-SC). Prior to chemical cross-linking, samples designated as dual-cross-linked (GelMA-DC) were additionally incubated at 5 °C for 1 h.

#### 2.3.2 Microemulsion Approach

To obtain samples with the microemulsion approach, initially, poly(ethylene oxide) (PEO, *M*_w_ = 300,000) (Sigma-Aldrich, UK) solution was prepared at the concentration of 1.6% (w/v) in PBS containing 0.1% (w/v) LAP. The solution underwent vigorous stirring at 40 °C for up to 1.5 h. To obtain hydrogel samples containing PEO, GelMA solutions were initially prepared at two different concentrations of 12.5 and 6.25% (w/v). The as-selected concentrations allowed for the incorporation of 20% (v/v) of PEO solution while ensuring that the GelMA remained constant, which equated to 10 and 5% respectively. The same principle was applied when incorporating 10% (v/v) PEO solution, using GelMA concentrations of 11.1% (w/v) for G10 and 5.6% (w/v) for G5, respectively. After the introduction of the PEO solution, the prepolymer solution was stirred for 10 s. Subsequently, the GelMA-PEO blend was then transferred to Teflon casting molds and cross-linked for 13 s (DC) and 60s (SC). Prior to this, dually cross-linked hydrogel samples were incubated at 5 °C for 1 h.

To ensure the clarity and transparency of the presented results, GelMA conditions tested in the present study are listed in Table 1, together with their simplified naming.

**Table 1:**
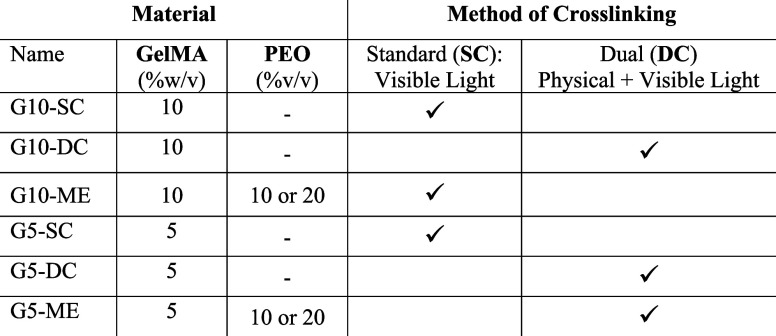
Composition of Materials Employed and Their Crosslinking Method

### 2.4 Mechanical Properties of GelMA

#### 2.4.1 Gelation Kinetics Assessment

Rheological measurements of G10 subjected to VL cross-linking and dual cross-linking were performed using a rheometer with a Peltier plate temperature system and a 20 mm serrated parallel plate accessory. The storage modulus and loss modulus of the samples were registered during the constant frequency of 1.6 Hz and sweeping temperature from 25 to 5 °C at a cooling rate of 3 °C/min. The respective hydrogel working solutions were incubated then up to 39 h. Young’s modulus was calculated based on the storage modulus of the sample, assuming a Poisson ratio of 0.5.

#### 2.4.2 Compressive Modulus Determination of GelMA Samples

The compressive modulus of GelMA-based samples was assessed using a dynamic mechanical analyzer (DMA) (Q800, TA Instruments, USA) at 37 °C with a 0.001 N preload force. Prior to analysis, cylindrical samples (*n* = 5) with a diameter of 6 mm and 1.6 mm in height were incubated in PBS overnight at 37 °C. Subsequently, specimens were subjected to a static compression test at a strain rate of 30%/min. The compressive modulus was calculated based on the slope of the linear region of the stress–strain curve in the range of 2–4% strain of the sample.

### 2.5 Swelling Behavior Determination

Hydrogel samples were dehydrated in increasing ethanol concentrations (Sigma-Aldrich, USA)—50, 70, 80, 90, and 2 × 100%, and left for overnight drying. To assess the stability of GelMA specimens, cylindrical samples (*n* = 5) were immersed in PBS solution and incubated for up to 72 h at 37 °C. At specified time points (every 10 min for the first hour, every 30 min for the second and third hours, every 60 min for the fifth hour, and then after 24, 48, and 72 h), the samples were collected, the excess water was gently wiped out, and the specimens were weighed again. The swelling degree (*H*) of each GelMA condition was evaluated using the following eq 2:
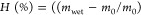
2where *m*_0_ is the initial weight of the dehydrated sample before immersion, and *m*_wet_ is the weight of the swollen sample measured at a fixed time point.

### 2.6 Porosity Calculations Using Microcomputed Tomography

Microcomputed tomography (micro-CT) analysis using the SkyScan 1172 system (Bruker, USA) was conducted to evaluate the porosity of GelMA-based samples. The GelMA samples were prepared following the procedure mentioned in Section 2.3. After fabrication, the samples at day 0 (D0) were stored at −80 °C and subsequently freeze-dried for 24 h. Samples designated as D1 underwent the same procedure after being immersed in DMEM-LG supplemented with 10% FBS and 1% PS for 24 h. The GelMA-based substrates were then subjected to micro-CT scanning with a source voltage of 40 kV and a source current of 250 μA. The resulting pixel size was 4 μm. During the scanning procedure, each sample was rotated by 180° with a step size of 0.15° and an exposure time of 80 ms.

### 2.7 Metabolic Activity of Cells during Cold Treatment

To evaluate the metabolic activity, human bone marrow-derived mesenchymal stem cells (hBMSC) were cultivated in low glucose DMEM supplemented with 10% fetal bovine serum (FBS) (EuroClone, Italy) and 1% penicillin-streptomycin (PS) (Gibco, USA). The cells from passage 8 were embedded (10^6^ cells/mL) in G10-SC and G5-DC and chemically cross-linked for 60 and 13 s, respectively. Cell-laden G5-DC specimens were physically cross-linked prior to visible light exposure, as previously optimized. Subsequently, the alamarBlue assay (Invitrogen, USA) was used to compare cell metabolic activity in G10-SC and G5-DC microarchitectures. In brief, cell-laden samples were introduced into 24-well plates filled with 900 μL of cell culture medium and incubated at 37 °C under 5% CO_2_. After 30 min of incubation, 100 μL of alamarBlue solution was added to the corresponding wells and incubated for 1h. The percentage of reduction of alamarBlue was then measured spectrometrically (FLUOstar Omega, BMG LABTECH, Germany) at 570 and 600 nm, according to the manufacturer’s specification. Subsequently, cell culture medium was removed, replaced with a fresh one and the samples were again incubated for up to 4 and 24 h. Results are presented as % of control; the 2D control involved seeding cells into a multiwell plate, followed by incubation under the same conditions as cells introduced to hydrogel substrates.

### 2.8 In Vitro Evaluation of Cell-GelMA Interactions

#### 2.8.1 Cell Culture

hBMSCs were expanded in a growth medium consisting of low glucose DMEM (ThermoFisher, USA), 10% FBS, and 1% PS, supplemented with 1 ng mL^–1^ human basic fibroblast growth factor 2 (hFGF) (Sigma-Aldrich, USA) until sufficient confluence was obtained. Cells from passage 5 were detached using trypsin–EDTA 1× (Gibco, USA) and embedded in GelMA-based constructs. Briefly, 4 × 10^3^ cells/mL were suspended in GelMA solutions (G5 and G10) and cross-linked as previously optimized. For PEO-containing specimens (G5-ME and G10-ME), cells were loaded in PEO solution and then transferred into GelMA. The growth medium was used up to day 4 of culturing, after which differentiation medium based on α-MEM-GlutaMAX TM (ThermoFisher, USA), 10% FBS, 1% PS, supplemented with 100 nM dexamethasone (Sigma-Aldrich, USA), 10 nM 1α, 25-dihydroxyvitamin D_3_ (Sigma-Aldrich, USA), 2 mM ß-glycerophosphate disodium salt hydrate (Sigma-Aldrich, USA), and 50 mM l-ascorbic acid 2-phosphate sesquimagnesium salt hydrate (Sigma-Aldrich, USA) was used to induce osteogenesis. The differentiation medium was changed every two to 3 days up to day 21. The cell-laden constructs (*n* = 3 for each condition) were incubated at 37 °C under 5% CO_2_. The specimens cultivated up to day 21 in a growth medium [named nondifferentiated (ND)] served as a control.

#### 2.8.2 Cell Viability Evaluation

Cell viability was determined using a LIVE/DEAD Cell Viability Kit (Invitrogen, USA, R37601) to investigate the response of cells encapsulated within different GelMA microarchitectures. At fixed time points of culture, that is, days 7, 14, and 21, samples were washed twice with PBS and immersed in 0.5 μL mL^–1^ calcein and 2 μL mL^–1^ ethidium homodimer. Calcein was used for staining live cells in green, while the ethidium homodimer was added for staining dead cells in red. Samples were incubated for 20 min at 37 °C and 5% CO_2_. Subsequently, samples were washed with PBS and imaged with a fluorescence microscope (Leica TCS SP8, Leica Microsystems, Germany) at wavelengths corresponding to fluorophores of interest. The number of alive (green, G) and dead cells (red, R) was quantified from fluorescence images using the counting algorithm of ImageJ (National Institute of Health, USA) on separate red and green channels of three different areas of independent samples (*n* = 3). The viability (%) was estimated using the equation *G*/(*G* + *R*) × 100.

#### 2.8.3 Characterization of Cell Morphology

Cell morphology was investigated by staining the cell actin filaments and nuclei. In brief, at days 7, 14, and 21 of incubation, GelMA constructs were washed with HEPES, and 4% paraformaldehyde was used to fix the specimens at 4 °C overnight. Subsequently, the specimens were washed with HEPES three times for 5 min each. Afterward, 0.3% (v/v) Triton X-100 in HEPES was added for 15 min, and samples were washed thrice. Hydrogel samples were then incubated in 1% (w/v) BSA in HEPES for 30 min and a 1:40 dilution of Alexa Fluor 488 Phalloidin (ThermoFisher, USA) in HEPES was added for 40 min at room temperature (RT). Afterward, specimens were washed and incubated in 1:1000 DRAQ5 (ThermoFisher, USA) solution diluted in HEPES for 10 min to visualize cells’ nuclei. Upon washing, samples were imaged under a confocal microscope (Leica TCS SP8).

#### 2.8.4 Alkaline Phosphatase Activity and DNA Quantification

Samples were removed from the medium and washed twice with PBS on days 7, 14, and 21, followed by freezing at −80 °C. To allow for GelMA disintegration and cell lysis, each sample was thawed and immersed in collagenase type II solution (Sigma-Aldrich, USA) (50 CDU in 200 μL of PBS) at 37 °C for 2 h, followed by its refreezing at −80 °C. To detect ALP activity in different microarchitectures of GelMA over the incubation period, the *para*-nitrophenylphosphate (*p*-NPP) method was used (Sigma-Aldrich, USA). A 50 μL portion of cell lysate was transferred into a 96-well plate, and an equal volume of diluted *p*-NPP was added to each well. The working solution was then incubated at room temperature for 45 min, and the absorbance was measured at 405 nm. The ALP activity was normalized by the DNA content. The DNA content was measured by a CyQUANT assay (Thermofisher, USA) using a DNA-based standard curve.

### 2.9 Statistical Analysis

Data are expressed as a mean ± standard deviation (SD). Two-way ANOVA followed by Tukey multiple comparisons and one-way ANOVA were performed using GraphPad Prism version 9.0 for Mac OS X, GraphPad Software, La Jolla California USA. Statistically significant values are displayed as *p value 0.0332 (*), 0.0021 (**), 0.0002 (***), and <0.0001 (****).

## 3 Results and Discussion

**Figure 1:**
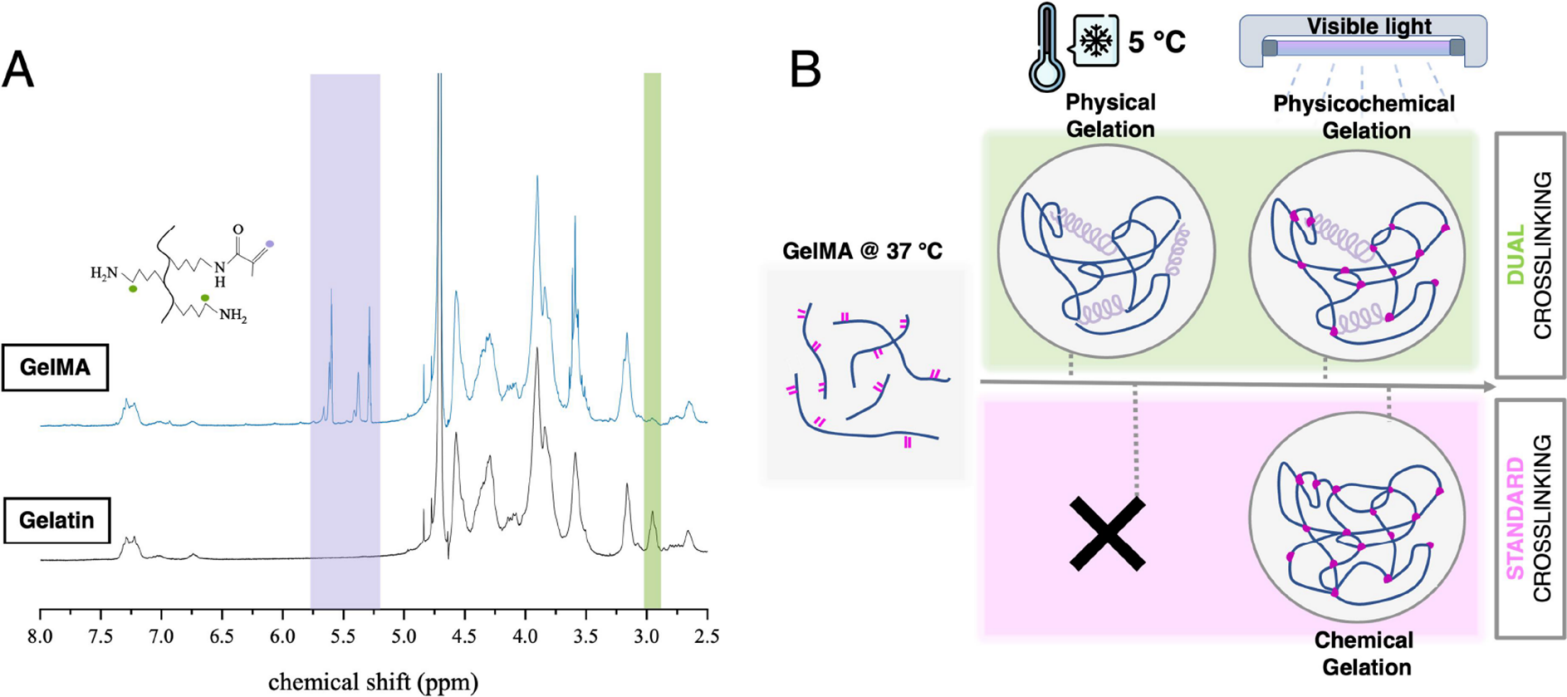
GelMA synthesis and cross-linking strategies: (A) ^1^H NMR spectra of unfunctionalized gelatin and GelMA in D_2_O. The peak around 2.9 ppm is associated with the lysine methylene proton on the gelatin backbone and was used to monitor the methacrylation reaction and determine the degree of functionalization; (B) schematic illustration of two strategies of GelMA cross-linking presented in the current work: (I) dual cross-linking based on physical gelation at 5 °C, followed by photoinduced cross-linking and (II) standard cross-linking based on the visible light exposure.

**Figure 2:**
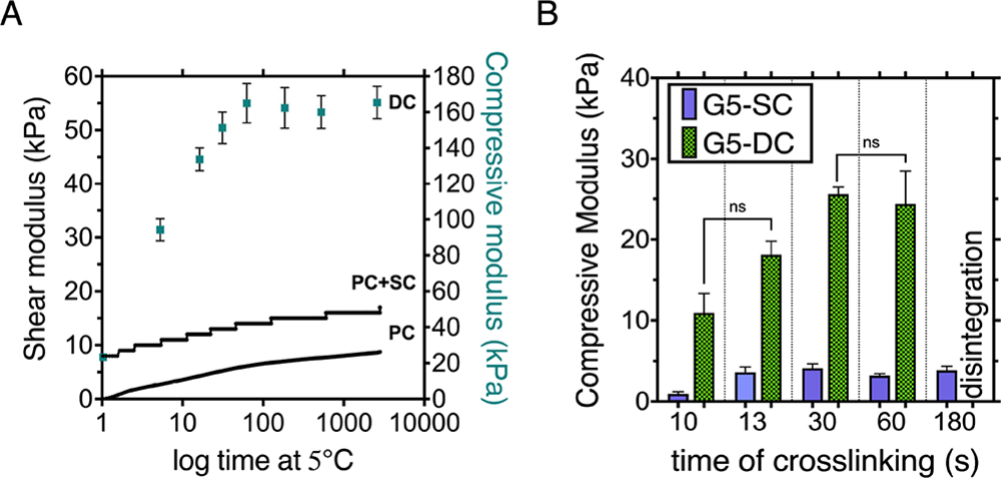
Optimization of GelMA cross-linking parameters to match the stiffness of osteoid tissue: (A) rheological and mechanical characterization of G10 exposed to physical cross-linking only (PC) and DC incubated up to 39 h at 5 °C, PC + SC—hypothetical value of dual-cross-linking plotted as physical gelation + offset value for chemically cross-linked G10; Poisson ratio of 0.5 assumed to convert moduli through *E* = 3·G; (B) compressive modulus of G5-SC and G5-DC samples incubated at 5 °C for 1 h followed by chemical cross-linking up to 180 s; significant difference was observed within various cross-linking times of G5-DC with the *p* < 0.0001 (****) apart from 10 vs 13 s (ns) and 30 vs 60 s (ns); the *p* < 0.0001 (****) was also noted between G5-SC and G5-DC (each cross-linking time).

Herein, the physical properties of GelMA were optimized to obtain four different osteoid-resembling hydrogel microarchitectures. We have decided to use GelMA hydrogel, which can be precisely and quickly tuned to promote bone formation in the long run.^36^ Additionally, for our bone tissue engineering application, we required hydrogels with a greater stiffness and increased resistance to degradation through hydrolysis. To meet these criteria, GelMA with a high degree of functionalization (DoF) following the established protocol^35^ was synthesized and subsequently quantified using ^1^H NMR spectroscopy (Figure 1A). The conversion of amine groups to methacrylamide was indicated with a peak around 2.9 ppm, corresponding to the lysine methylene proton on the gelatin backbone, having diminished. The ratio of integrals of this peak at 2.9 ppm between the gelatin and GelMA spectra, after normalizing to the phenylalanine peak at 7 ppm, was used to quantify the DoF for the GelMA macromonomers at 79 ± 4%. Additional peaks were observed around 5.5 ppm, corresponding to the acrylic protons on the methacrylamide and methacrylate groups, while residual signals on the GelMA spectrum showed similar chemical shifts and intensities to those of the gelatin, thus indicating that the primary structure of the gelatin molecule had been maintained during the reaction. While following a frequently employed protocol for assessing the DoF in GelMA,^33,37,38^ future considerations for unbiased quantification could involve the utilization of a reference molecule such as 3-(trimethylsilyl)propionic-2,2,3,3-d4 acid sodium salt (TMSP),^39^ potentially offering more accurate results. Two different strategies of GelMA cross-linking (Figure 2B) were proposed to obtain a stiffness like collagen-rich osteoid, which is estimated to be 27 ± 10 kPa.^40^ The first cross-linking strategy involved immobilizing the polymer chains of GelMA through exposure to visible light. As this method is widely used in tissue engineering, we have decided to classify it as a standard cross-linking (SC) and treat it as a reference for further material optimization. Granting that light-induced cross-linking is a rapid process to obtain defined properties of hydrogels, the challenge lies in creating robust structures that simultaneously enhance cell viability and degrade at a predetermined rate. Moreover, the biodegradation rate of such hydrogels is reduced, making them less attractive for tissue engineering applications. However, the compromise between what is required and what is possible must always be considered. Indeed, studies have reported that GelMA hydrogels with low monomer concentrations (approximately 5%) and lower degrees of substitution (DS) are more favorable for supporting embedded cell proliferation, sprouting, and network formation, especially for vascular cells^41,42^ However, the problem arises when stiff constructs are needed to be obtained. One of the methods of creating stiff GelMA constructs is locking in the complex structures and hydrogel bonds permanently by combining thermal gelation and chemical cross-linking. Thus, we have decided to implement a two-stage cross-linking, that is, physical gelation followed by visible light exposure (Figure 1B) to easily tune the microarchitecture of GelMA without hampering their initial stiffness, simultaneously creating a more appropriate environment for bone cells’ survival and maturation. The method of reversible physical cross-linking was previously explained as the formation of helical chains in the structure of GelMA^26,43^ Weak hydrogen bonds are formed between gelatin and water, creating a disorganized net of partial helices and trapped water. To effectively lock in the structure of the hydrogel, photoinitiated cross-linking was eventually applied. Having this in mind, it was believed that with the use of a low concentration of GelMA and reduced chemical cross-linking time, the stiff constructs might be obtained when neither the prolonged exposure to light (≥60 s) nor the high (≥10%) concentrations of GelMA are used.

### 3.1 Effects of Cold Incubation Prior to VL Cross-Linking on the Mechanical Properties of GelMA

The stiffness of 10% (w/v) GelMA gels increased by a factor of 7 when the precursor solution was incubated for 1 h or more (up to 39 h) before VL cross-linking (i.e., dual cross-linking) compared to direct VL cross-linking of the precursor solution without cold incubation: 164 ± 9 vs 23 ± 1.4 kPa. The kinetics of physical cross-linking were assessed by conducting rheological characterization of a 10% GelMA solution incubated at 5 °C for almost 50 h and following the increase in shear modulus over time (Figure 2B, curve labeled PC for physical cross-linking). To understand if the effect of physical gelation is additive or synergistic to chemical cross-linking through VL exposure, we plotted the time-dependent increase of shear modulus that resulted from physical cross-linking at an offset of the modulus obtained by chemical cross-linking for G10 (Figure 2A, curve PC + SC). To enable this comparison between shear and compressive moduli, a Poisson ratio of 0.5 was assumed. It is evident that the compressive modulus of DC gels (Figure 2B scatter plot DC) is much higher than the combined effect of SC and physical cross-linking, demonstrating the synergistic effect of the two types of cross-links being in agreement with the literature.^26^ Despite physical cross-linking still increasing up to 49 h incubation as evidenced, Young’s modulus of G10-DC gels increased with time only over the first hour, after which it reached its plateau value. In light of this, the choice of GelMA incubation conditions proved to be relatively uncomplicated. Our findings indicate that a 1 h incubation at 5 °C is sufficient to reach the desired stiffness.^44,45^

**Figure 3:**
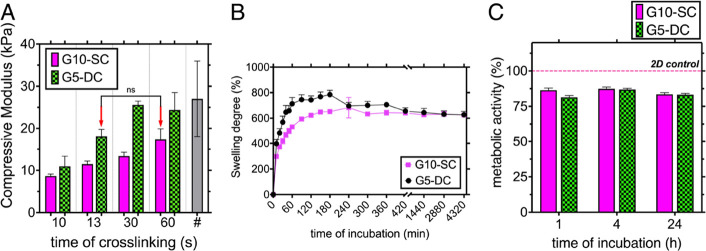
Characterization approaches of G10-SC and G5-DC: (A) influence of the time of chemical cross-linking on the compressive modulus of G10-SC and G5-DC detected using DMA; #—literature-based compressive modulus of osteoid tissue; (B) swelling degree of the samples fabricated using different strategies of cross-linking and incubated in PBS up to 72 h at 37 °C; (C) metabolic activity of hBMSCs embedded into G10-SC and G5-DC determined by an Alamar Blue assay; metabolic activity is presented as a % of reduced resazurin by hBMSCs cells vs 2D control.

Following optimization of the incubation time, the photo cross-linking time for G5-DC was studied and compared to light-exposed G5-SC (Figure 2B). As for the 10 wt % gels, the 1 h of cold incubation prior to VL cross-linking caused an 8x increase in stiffness. Furthermore, the stiffness of the G5-DC hydrogels demonstrated a notable increase in response to VL exposure. Specifically, the compressive modulus elevated from 11.91 ± 1.2 to 24.39 ± 4.12 kPa when exposed for 10 and 60 s, respectively. Conversely, the stiffness of the G5-SC constructs exhibited minimal fluctuations consistently measuring around ∼3.12 kPa across all tested conditions. Moreover, it was found that exposing G5-DC samples to 180 s of VL-cross-linking resulted in the formation of very brittle hydrogel structures which disintegrated upon manipulation. This finding was believed to be the result of the formation of an exceedingly high number of chemical cross-links, which prevents the extension of polymer chains in response to an applied load.^46^ To achieve comparable stiffnesses to G10-SC, G5-DC samples were cross-linked with VL for 13 s to give a compressive modulus of 18.12 ± 1.66 kPa (Figure 3A).

### 3.2 Advancing GelMA Cross-Linking Strategies for the Generation of Osteoid-like Stiffness

To obtain hydrogels of different microarchitectures but matched stiffness in the range of osteoid tissue, the cross-linking time was adjusted for both G10-SC (60 s) and G5-DC (13 s) gels (Figure 3A). To validate the effect of dual cross-linking on the physical properties of GelMA, the swelling degree of G5-DC was compared to the G10-SC. It was assumed that the use of a lower concentration of GelMA macromonomer would result in accelerated degradation, thereby closely resembling the natural remodeling conditions of bone tissue. In this study, it was found that G5-DC compared to G10-SC showed a higher initial water uptake over 180 min (Figure 3B), when constructs were incubated at 37 °C (784.7 ± 68.5% vs 652.03 ± 15.9% for G5-DC vs G10-SC), which is in agreement with the literature.^47^ Surprisingly, this trend was not observed across the whole incubation period, where after 7 h of the samples being immersed in PBS, specimens of G5-DC and G10-SC showed similar swelling behavior, that is, 655.68 ± 51.90 and 640.92 ± 14.59%, respectively. This swelling behavior was found to be affected considerably by shrinkage of G5-DC samples (∼26% compared to G10-SC) after immersion in PBS at 37 °C.

We compared the metabolic activity of the relevant cell type human bone marrow-derived mesenchymal stem cells hBMSCs encapsulated in GelMA platforms with different concentrations and cross-linking strategies (Figure 3C). hBMSCs were chosen as they have the ability to differentiate into osteoblasts and hence contribute to the process of bone tissue reproduction and growth.^48^ Therefore, it was essential to prove that hBMSCs will remain alive once in contact with the material during two-stage cross-linking. The metabolic activity of cells was assessed by measuring the percentage of resazurin reduced at 1, 3, and 24 h after encapsulation in G5-DC and G10-SC samples. During the first hour of incubation, the percentage of reduced resazurin was ∼81 and ∼86% for G5-DC and G10-SC, after which the metabolic activity of cells stabilizes over the incubation period reaching a final value of 83.2 ± 1.2 and 83.5 ± 1.1% for G5-DC and G10-SC, respectively, at day 1 of incubation. This corresponds to the results obtained during water absorption measurements, where the tendency of water uptake of G5-DC and G10-SC samples also stabilizes after 24 h of incubation. The evaluation of metabolic activity of cells encapsulated within G5-DC and G10-SC demonstrated that the fabrication and cross-linking strategies did not exert any toxicity on hBMSCs. The slightly reduced values observed in the hydrogel samples, in contrast to those in 2D, may be attributed to the differences between 2D and 3D environments. In the 2D cell configuration, more rapid proliferation could be observed, resulting in higher value intensities. Furthermore, the 2D systems exhibit a faster exchange of resazurin/resorufin when compared to the 3D gel structures. Additionally, it is possible that the hydrogels retained some of the resorufin, which could account for the lower readings of the metabolic activity.

**Figure 4:**
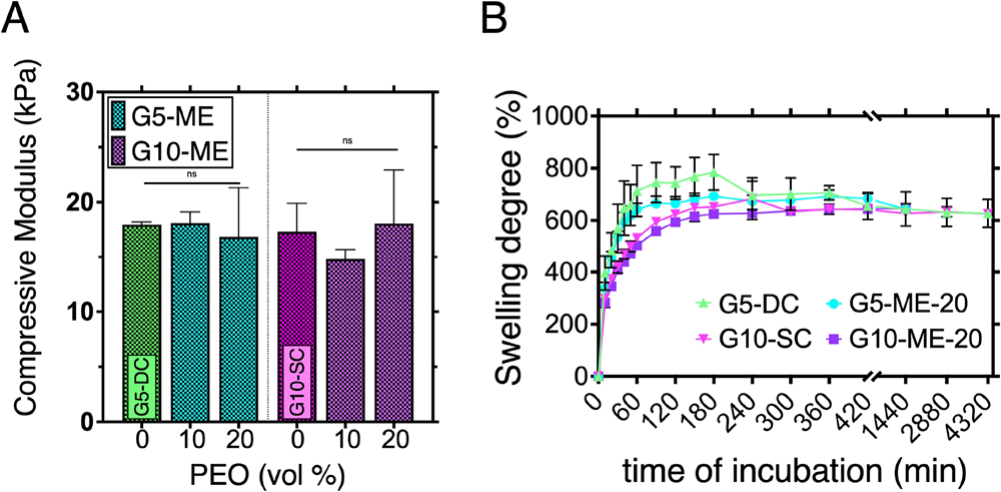
Characterization of GelMA constructs with PEO addition/removal: (A) compressive modulus of G5- and G10-based specimens; samples without PEO addition served as a control—G5-DC and G10-SC; (B) water absorption determination of G5- and G10 samples with 20 vol % addition after 24 h of incubation in PBS at 37 °C compared to pristine G5-DC and G10-SC.

Once cross-linking strategies were optimized, a microemulsion approach to obtain structures of G5-DC and G10-SC which contain PEO as a porogen was introduced. As was previously discussed, PEO enables adjustable micropore formation in the structure of GelMA and, thus, could provide the opportunity to enhance cell spreading and tissue formation.^34^ To achieve a hydrogel structure with a greater hierarchical pore arrangement, the concentration of GelMA prepolymer solutions was adjusted accordingly to maintain the desired concentrations of G5 and G10 following the addition of 10 and 20% (v/v) PEO solution. Consequently, specimens were cross-linked forming constructs, which we will designate in this paper as G5-ME (two-stage cross-linking) and G10-ME (exposed to VL). The incubation of specimens in PBS for 24 h at 37 °C was assumed to enable PEO removal from the structure of the hydrogels. Prior to the detection of morphological changes in the structure of hydrogels upon PEO removal, we evaluated G10-ME and G5-ME in terms of their stiffness. It was believed that the removal of PEO would cause a decrease in the compressive modulus of specimens due to an increase in porosity as was presented in the work of Ying et al.^33^ Thus, we have decided to focus on PEO addition up to 20 vol % into the prepolymer solution not to observe a severe drop in their initial stiffness after cross-linking. Surprisingly, the results were in contrast to those shown in the above-mentioned publication, where we observed that with an increased concentration of PEO, the compressive modulus did not change significantly for G5-ME and G10-ME—remaining at ∼18 kPa regardless of the percentage of PEO (Figure 4A). The differences between our results and those presented in the literature may be due to different GelMA concentrations. In our experiments, the GelMA prepolymer solution was concentrated before adding PEO solution, whereas the approach presented by Ying et al. focused on GelMA solution dilution.^28^ Even though noticeable changes in material stiffness were not observed, the ratio of weight percentage of PEO to GelMA was relatively small, that is, ∼6 and 3 wt % for G5 and G10, respectively, so it should not be surprising that the differences may not have been visible at first glance.

To investigate the microarchitecture of the GelMA hydrogels developed in this work, water absorption up to 72 h was studied on samples incubated at 37 °C (Figure 4B). This experiment was used to make predictions on the porosity of the hydrogel constructs, where it was assumed that a greater water uptake correlated with an increased PEO removal from the hydrogel, could result in a high porosity. The results show a lower degree of water uptake for G5-ME and G10-ME compared to G5-DC and G10-SC, suggesting that there could be insufficient removal of PEO from the structure of the hydrogels. It was also observed that the swelling degree after 24 h of incubation stabilizes, reaching ∼625%.

**Figure 5:**
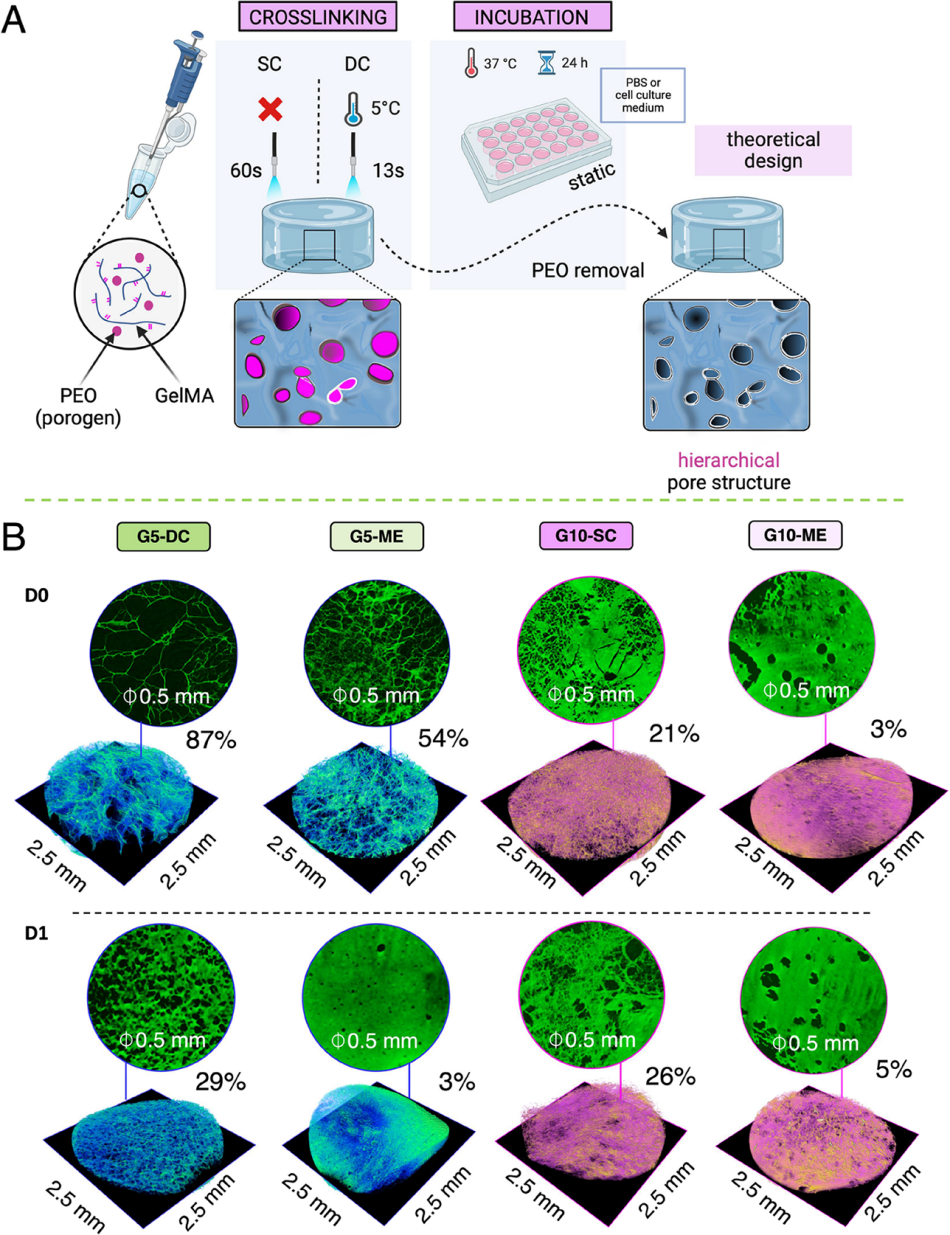
Quantification of porosity changes in freeze-dried G5- and G10-based samples: (A) schematic workflow of intended PEO removal from GelMA structure during 24 h incubation; (B) micro-CT scan reconstructions at day 0 (D0, samples were fabricated according to the cross-linking approach and then freeze-dried) and day 1 (D1) of incubation in DMEM-LG medium supplemented with 10% FBS and 1% PS. The images are shown as a region of interest (ROI) of the sample with *x* and *y* equal to 2.5 mm.

To further investigate whether PEO remains in the structure of the hydrogels after 24 h of incubation in PBS or cell culture medium, optical images of GelMA-based samples were taken (Figure S1). Samples containing 20 vol % PEO globules were opaque compared to transparent specimens of G5-DC and G10-SC just after its fabrication (D0). Significant removal of PEO from the GelMA structure after 24 h of incubation was not observed, as initially intended (Figure 5A) Micro-CT analysis was performed to evaluate the porosity of the samples. The results presented in Figure 5B indicated a decrease in porosity with increasing GelMA concentration. Specifically, the porosity was 87% for G5-DC and 21% for G10-SC at D0. The addition of PEO to G5-DC and G10-SC, resulting in G5-ME and G10-ME, further reduced the porosity by approximately 38 and 86%, respectively, compared with the pristine samples at D0. However, it should be noted that the incomplete removal of PEO was evident as the porosity for G5-ME reached only 3%. The porosity for G10-ME samples was slightly higher but not significantly different from D1. Overall, the optical and micro-CT analysis confirmed together with FTiR and TGA spectra (Figures S4 and S5, respectively) the presence of PEO in GelMA-based hydrogels even after 24 h of incubation, indicating the challenges associated with complete PEO removal from the microarchitecture.

### 3.3 Evaluation of Biological Properties of GelMA-Based Constructs

**Figure 6:**
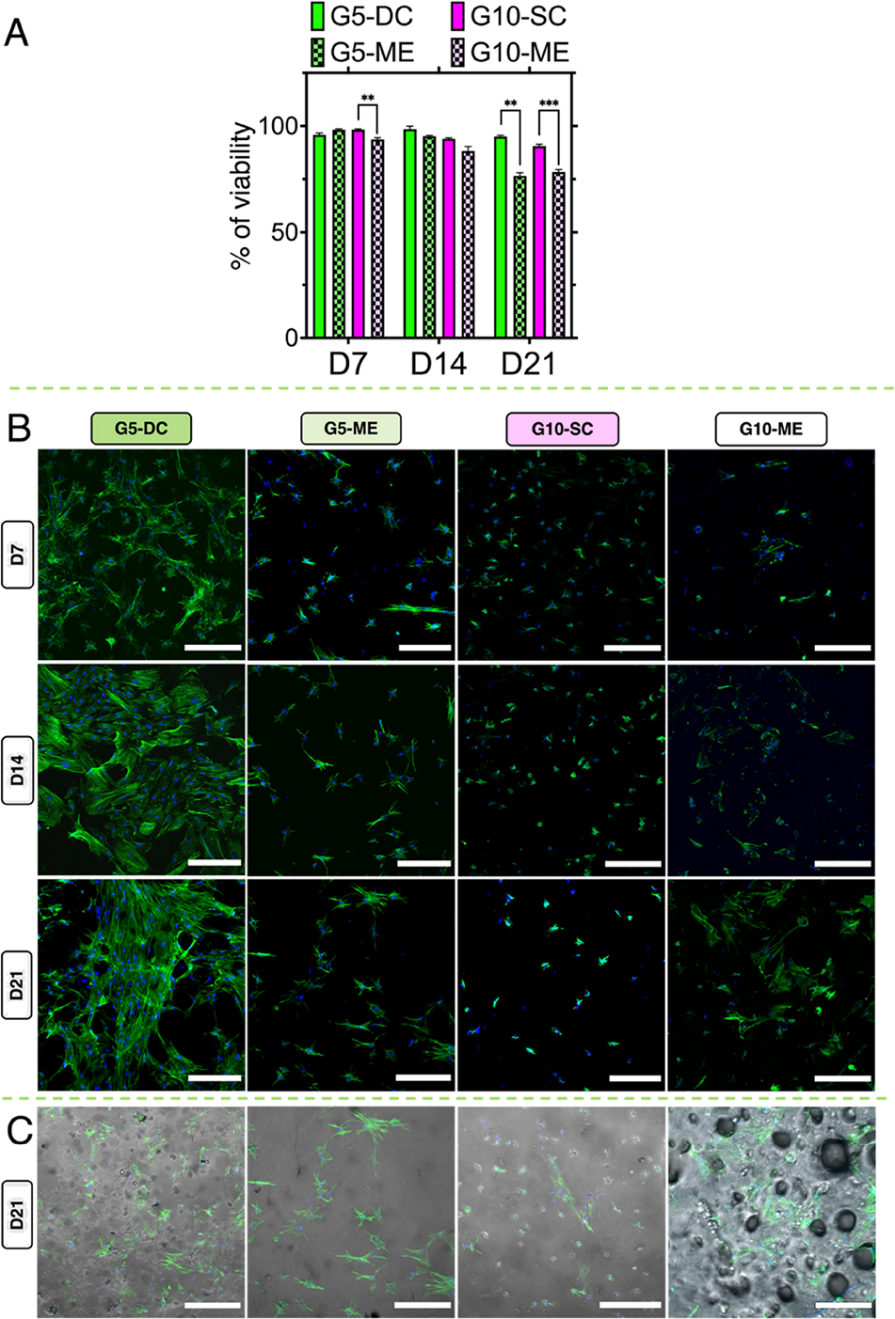
Biological evaluation of GelMA hydrogel constructs. (A) hBMSC cell viability over 3 weeks of incubation in osteogenic medium, (B) cell morphology visualization with actin (green) and cell nuclei (blue) staining, (C) bright-field images of G5- and G10-based samples at day 21 (D21) of incubation in osteogenic medium. Scale bar 300 μm; *p* = 0.0021 (**), < 0.0001 (***).

hBMSC was selected to cellularize the microarchitectured GelMA due to its in vitro osteogenic differentiation potential. Cell-laden constructs were tested for up to 21 days (Figures 6 and S2). Cell viability was evaluated by live/dead assay and all tested conditions exhibited high cell viability (>90%) at day 7 (Figure 6A). Cells encapsulated in G5-based samples did not show any significant difference between the two investigated groups (i.e., G5-DC and G5-ME) up to 2 weeks of culture (day 14). Cell adaptation over the culture time (i.e., up to day 21) was detected in G5-DC structures, with no significant changes in the viability, thus speculating the positive influence of the dual cross-linking approach on the proposed hydrogel. Additionally, the structural integrity of G5-based samples was well-preserved throughout the cultivation period, as shown in Figure S3. However, a gradual degradation of G5-DC was evident, with a transition toward a more transparent-like structure at day 21 compared to day 7, highlighting the ongoing remodeling process of the hydrogel construct. Unfortunately, increased proliferation of hBMSCs embedded in G5-ME was not observed as it was anticipated by Ying et al., where the viability of HepG2 loaded into PEO-containing GelMA increased by 7% compared to the pristine samples at day 7.^33^ In our case, the viability of porous G5-ME samples significantly decreased compared to that of the G5-DC counterpart at day 21 (76.52 vs 95.07%. respectively), likely due to the low adaptability of cells to the nonremoved PEO phase. On day 21, G5-ME showed a 21.73% lower cell survival than that on day 7. A similar trend was observed in the case of G10-ME, where cell survival displayed a 15.35% drop in the same time range (from day 7 to day 21). G10-SC did not show any change in cell viability over the culture time, although a significant difference in cell viability was observed when comparing G10-SC to G10-ME at day 7 and day 21 (98.28 vs 93.74% and 90.52 vs 78.39%, respectively). These results do not provide support for the hypothesis that the PEO phase promoted cell growth and enhanced the biocompatibility of the GelMA hydrogel after a 3-week culture. Such observations contradict previous studies that showcased the viability of the porous GelMA-PEO hydrogels. As previously mentioned, the incomplete removal of PEO globules in our case can be attributed to the dense network structure of GelMA, resulting in globules entrapment within the shrunk G5-based substrates.^28^

Furthermore, cell spreading and morphology were also evaluated over 3 weeks of culture time by actin staining to understand the influence of the different fabrication and gelation methods on the cytoskeleton structure (Figure 6B). In G5-DC, cells freely spread in all directions within the hydrogel constructs up to day 21, as confirmed from the elongated and nonoriented actin filaments. At day 21, cells formed a more interconnected intercellular network of fused cytoskeletons, showing that hBMSC morphology is positively affected by the dual-cross-linking method. Culture of hBMSCs in G5-ME, G10-ME, and G10-SC (which had a comparable stiffness to G5-DC) hydrogels resulted in limited cell proliferation and elongation without notable spreading over time. This supports the hypothesis that tuning stiffness alone is not sufficient for designing hydrogels that facilitate neotissue maturation; microarchitecture is just as an essential factor if not more important. Furthermore, it is possible to speculate that PEO removal in porous structures (i.e., G5-ME and G10-ME) did not fully occur (Figure 6C), thus hampering the creation of void-forming hydrogels and, in turn, osteoid tissue formation. Similar results were obtained for G10-SC, where the high GelMA macromonomer concentration in the hydrogel resulted in a gel with low permeability, limiting both PEO removal and cell maturation.

**Figure 7:**
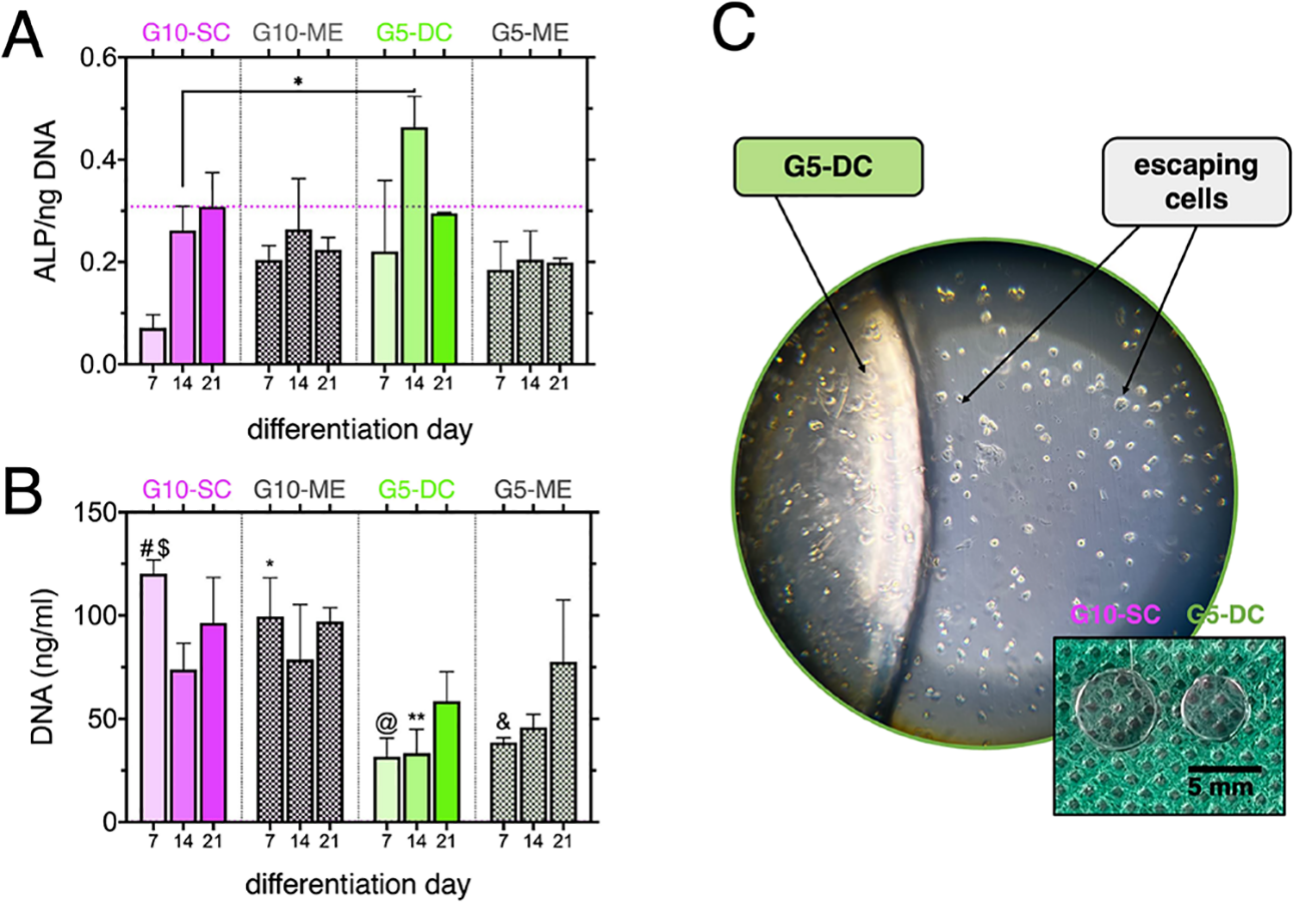
ALP expression and DNA content during the in vitro culture of G5- and G10-based samples: (A) normalized alkaline phosphatase (ALP) activity to DNA content over 3 weeks of incubation in osteogenic medium, (B) single data set representing DNA levels used for ALP normalization, (C) image of shrunk G5-DC sample after immersion in cell culture medium for 24 h, which revealed the presence of escaping cells from the structure of the hydrogel; #—significant difference compared to D7-G5DC, D7-G5ME and D14-G5DC (*p* 0.0021); $—compared to D14-G5ME (*p* 0.0332); *—D7-G5DC, D7-G5ME, D14-G5DC; (*p* 0.0332); @—D21-G10SC, D21-G10ME (*p* 0.0332); &—D21-G10SC, D21-G10ME (*p* 0.0332); **—D21-G10SC, D21-G10ME (*p* 0.0332).

Comparison of the normalized alkaline phosphatase (ALP) activity to the DNA content was conducted to provide valuable insights into the osteogenic potential and cellular behavior within different hydrogel materials (Figure 7). In our study, four GelMA-based hydrogels were cultivated in osteogenic medium for up to 3 weeks, while the samples incubated in nonosteogenic medium served as a negative control (Figure S6). Throughout the cultivation period, all hydrogels exhibited an increase in ALP activity, indicating successful osteogenic differentiation of the cells within the hydrogel materials (Figure 7A). Moreover, nondifferentiated hydrogel samples showed 2-fold reduction in ALP activity compared to those undergoing osteogenic differentiation (Figure S6). Notably, the hydrogel without PEO addition, G5-DC, demonstrated the highest ALP/DNA content up to day 21. The ALP production was particularly prominent at day 14 (0.46 ± 0.06) for G5-DC, followed by a slight decrease to approximately 0.3 at day 21, which is consistent with previous findings.^36^ In comparison, the ALP/DNA content for G10-SC was 0.26 ± 0.3 on day 14 and 0.31 ± 0.07 on day 21. Interestingly, the DNA content (Figure 7B) at the same time points was significantly lower for G5-DC, approximately 45 and 39% at day 14 and day 21, respectively, compared to G10-SC. This discrepancy suggests the possibility of cells escaping from the shrunk architecture of the G5-DC samples (Figure 7C). Notably, the DNA content increased for the dually cross-linked samples (G5-DC and G5-ME), indicating ongoing cell proliferation within the microarchitecture. As anticipated, the addition of PEO to the hydrogel substrates did not significantly enhance osteogenic differentiation.

## 4 Conclusions

This study aimed to optimize the physical properties of GelMA hydrogels to create osteoid-like microarchitectures for bone tissue engineering applications. GelMA was chosen due to its tunability and ability to promote bone formation. Two cross-linking strategies were proposed: standard cross-linking (SC) using visible light and a two-stage cross-linking approach combining physical gelation and visible light (VL) exposure.

By reducing the GelMA concentration from 10 to 5% and implementing physical gelation prior to VL, we have achieved a stiffness comparable to an osteoid matrix (27 ± 10 kPa).^40^ We demonstrated that physical gelation did not compromise cell viability during 1 h of incubation at 5 °C, and the DC strategy promoted bone tissue formation as evidenced by alkaline phosphatase (ALP) measurements. Furthermore, to establish hierarchically porous GelMA constructs for enhanced bone formation, we proposed the addition of 20 vol % PEO into the hydrogel structure. However, the PEO was confirmed to be locked in the dense structure of the polymer network and insufficiently removed due to both significant shrinkage of DC-based samples and porosity reduction from 87 to 29% for G5-DC just 1 day postfabrication. Similar trends were observed in GelMA 10% constructs exposed to VL.

The viability of human bone marrow-derived mesenchymal stem cells (hBMSCs) encapsulated in GelMA platforms was not inhibited by physical gelation, and cells remained viable throughout the incubation period. Moreover, hBMSCs in dual-cross-linked gels showed an increased ability to spread, formed intercelluluar connections, and remodeled the matrix, alongside increased expression of the early osteogenic marker alkaline phosphatase compared to those in standard-cross-linked GelMA of the same stiffness. This underlines the importance of the microarchitecture in hydrogel-based tissue engineered scaffolds.

Micro-CT analysis confirmed that the porosity of the constructs can be reduced by increasing GelMA concentration and in turn adding poly(ethylene oxide) (PEO). Surprisingly, the addition of PEO as a porogen did not allow for the formation of hydrogel structures with hierarchical pore arrangements. During the incubation period, it was observed that PEO globules remained trapped within the structure of the 5% GelMA hydrogels. This was due to the physical cross-linking, which effectively locked in the PEO globules within the hydrogel matrix. As a result, the hydrogel porosity was hindered on the first day of incubation. Furthermore, when GelMA was used with a higher concentration (10%), the dense polymer network prevented the complete removal of the porogen material. Only the untrapped globules on the surface of the hydrogel were removed, while those embedded within the structure remained enmeshed.

These observations shed light on the challenges associated with influencing the porosity of GelMA hydrogels, specifically when using PEO as a porogen. The physical cross-linking and the dense polymer network of GelMA at higher concentrations can impede the complete removal of porogens, leading to reduced porosity within the hydrogel structure.

Overall, this study highlights the potential of coordinated physical gelation and chemical cross-linking to produce hydrogels with substantially enhanced mechanical properties and high cell viability—conditions that favor bone tissue formation. These findings contribute to the development of biomimetic hydrogel scaffolds for bone tissue engineering applications.

## Supporting Information Available

Additional experimental details of qualitative observations of structural stability of GelMA-based samples, infrared spectra and thermogravimetric analysis of freeze-dried constructs, and APL/DNA content of GelMA hydrogel samples (PDF)
