# Supplementary material for: Tuning Physical Properties of GelMA Hydrogels through Microarchitecture for Engineering Osteoid Tissue
Source: Biomacromolecules. 2023 Dec 16;25(1):188–99. doi: 10.1021/acs.biomac.3c00909 (PMC11106746; doi:10.1021/acs.biomac.3c00909)
Supplement: Supplementary file 1 — bm3c00909_si_001.pdf [file bm3c00909_si_001.pdf]

Supporting Information

Tuning physical properties of GelMA hydrogels through microarchitecture for engineering osteoid tissue

Ewa Walejewska[1,2], Ferry P.W. Melchels[3], Alessia Paradiso[1], Andrew McCormack[3], Karol Szlazak[1], Alicja Olszewska[1], Michal Srebrzynski[4,5], Wojciech Święszkowski[1*]*

[1] Faculty of Materials Science and Engineering, Warsaw University of Technology, Woloska 141, 02-507 Warsaw, Poland
[2] Centre for Advanced Materials and Technologies CEZAMAT, Warsaw University of Technology, Poleczki 19, Warsaw, 02-822, Poland
[3] Institute of Biological Chemistry, Biophysics and Bioengineering, Heriot-Watt University, Edinburgh, Scotland, UK
[4] Department of Transplantology and Central Tissue Bank, Medical University of Warsaw, Chalubinskiego 5, 02-004 Warsaw, Poland
[5] National Centre for Tissue and Cell Banking, Chalubinskiego 5, 02-004 Warsaw, Poland

* Corresponding authors: ewa.walejewska.dokt@pw.edu.pl
wojciech.swieszkowski@pw.du.pl

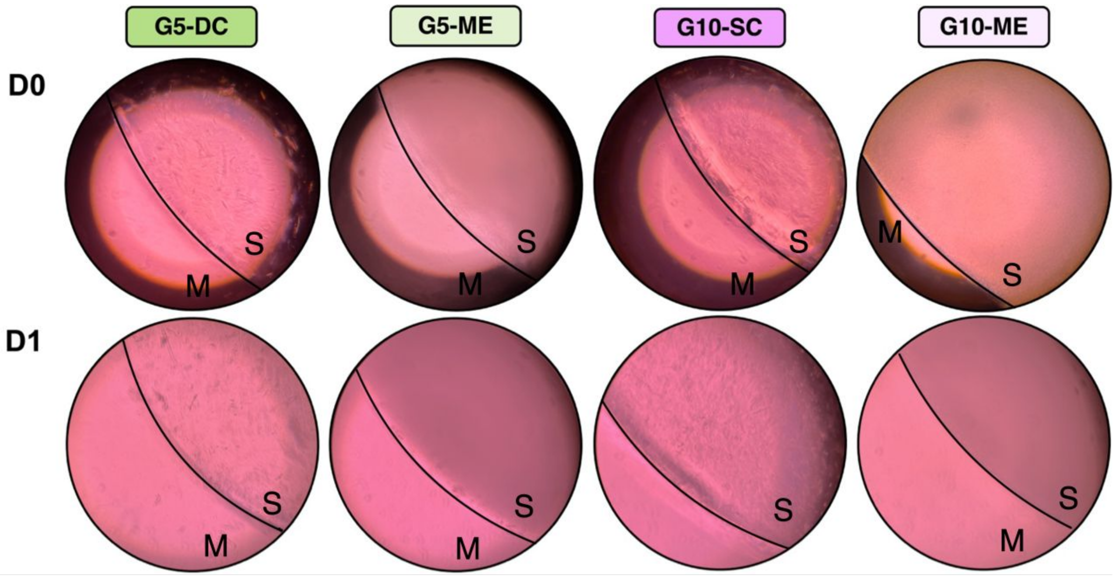

Figure S1 Optical images of GelMA-based samples depicting the differences in the transparency between tested conditions up to day 1 of incubation. *The samples containing PEO particles were opaque compared to transparent specimens of G5-DC and G10-SC. This supports our assumption, that significant removal of PEO from GelMA structure was not observed. Samples were incubated in cell culture medium (without embedded cells), where M stands for DMEM LG medium supplemented with 10% fetal bovine serum and 1% penicylin-streptomycyn; and S – immersed specimen.*

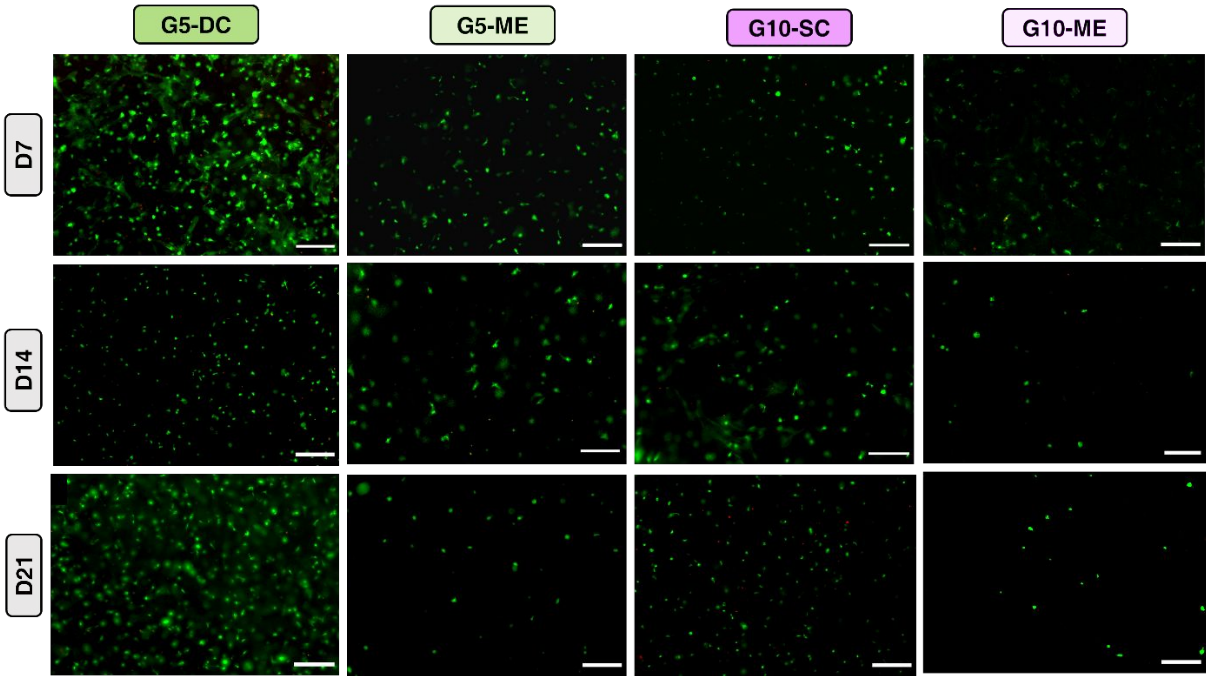

Figure S2 Live/dead images of GelMA-based samples during three-week incubation period in osteogenic medium. *Fluorescent images enabled to calculate the viability of the constructs using algorithm of ImageJ (National Institute of Health, USA) on separate red and green channels of three different areas of independent samples (n = 3). Scale bar 400 um.*

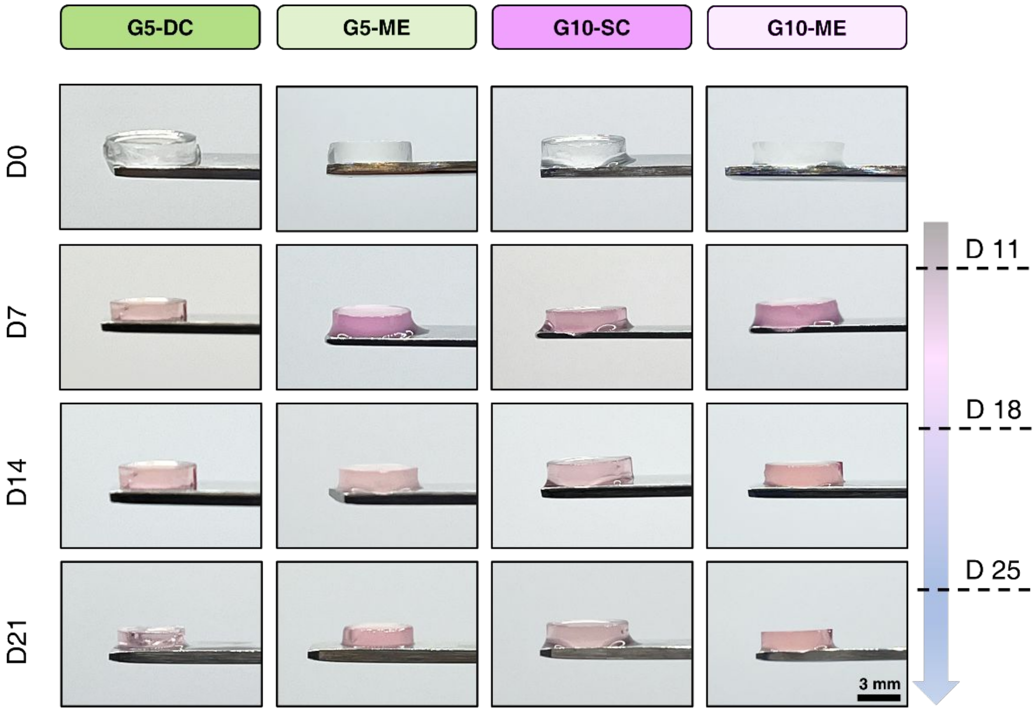

Figure S3 Structural stability of hBMSCs-loaded GelMA-based samples during three-weeks incubation in osteogenic medium*. The cultivation process involved a four-day incubation in a non-differentiation medium, followed by a 21-day exposure to osteogenic factors.*

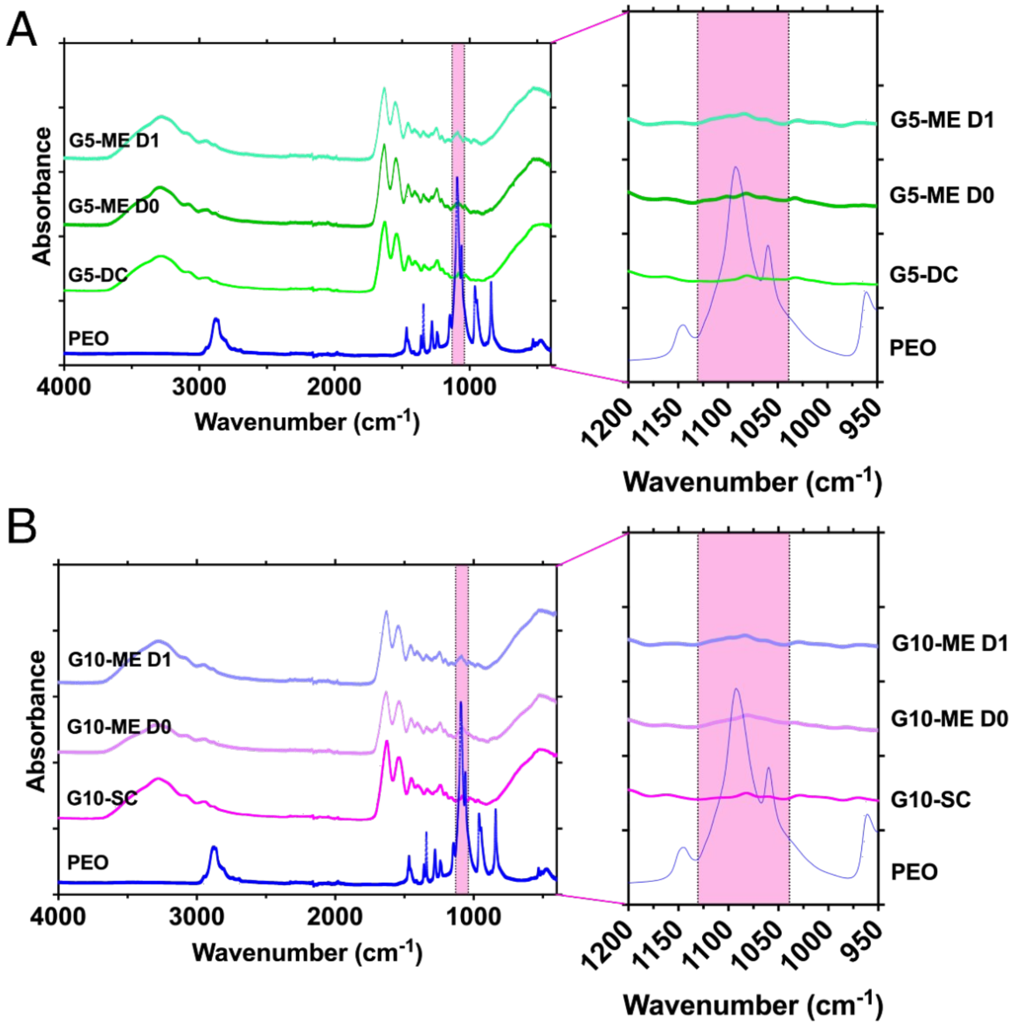

Figure S4 Infrared spectra of freeze-dried A) G5-based and B) G10-based *specimens were acquired using a Fourier transform infrared spectrophotometer (Nicolet 8700 FTIR, ThermoScientific). Measurements were performed using FTIR ATR over a range of 4000–400 cm−1. For clarity in presentation, the plots depicting absorbance were systematically shifted by n+0.5 for each material. The absorption peak, highlighted in pink, starting at the wavenumber of 1130.56 cm-1 and ending at 1038.96 cm-1 corresponds to C-O-C stretching mode, and it is visible in G5- and G10-based samples. Pristine G5-DC and G10-SC samples exhibited a more subdued peak, deviating from the hydrogels containing PEO particles. The absorbance peak shape of G5-ME and G10-ME remained unchanged even after a day of incubation in PBS, resembling the peak observed immediately after sample fabrication. This observation raises the possibility of PEO removal from the hydrogel structure being incomplete.*

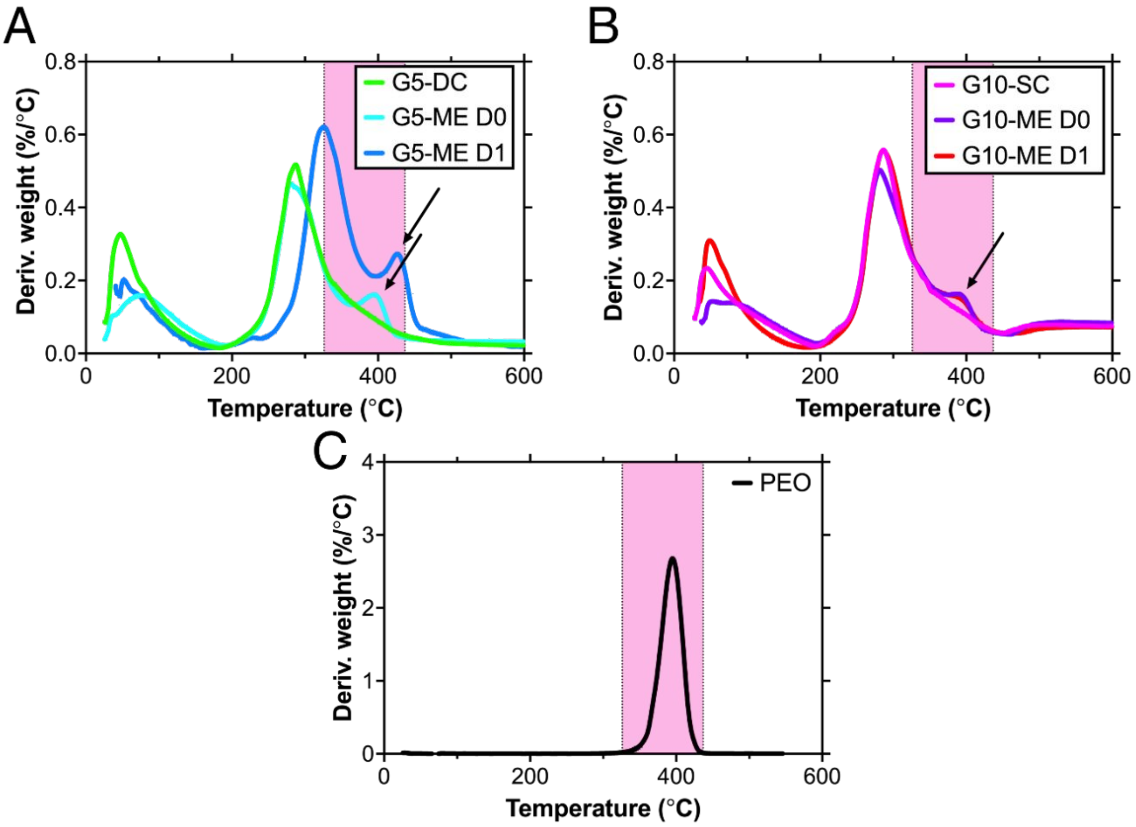

__Figure S5 Thermogravimetric analysis (TGA) plots for freeze-dried__ A) G5-based and B) G10-based hydrogel samples together with C) PEO powder. The results depict the derivative of weight (%/°C) as a function of temperature. The TGA procedure was performed using thermogravimetric analyzer Q5000 (TA Instruments, USA). Each specimen was heated from 25°C to 600 °C at a rate of 10 ° C/min under nitrogen atmosphere (flow of 25 ml/min). The most prominent peak, starting ~ 326 °C and ending ~ 436°C, corresponding to PEO, is highlighted in the structures of G5-ME and G10-ME at D0 and D1 of incubation. This finding lends support to the hypothesis of incomplete PEO removal.

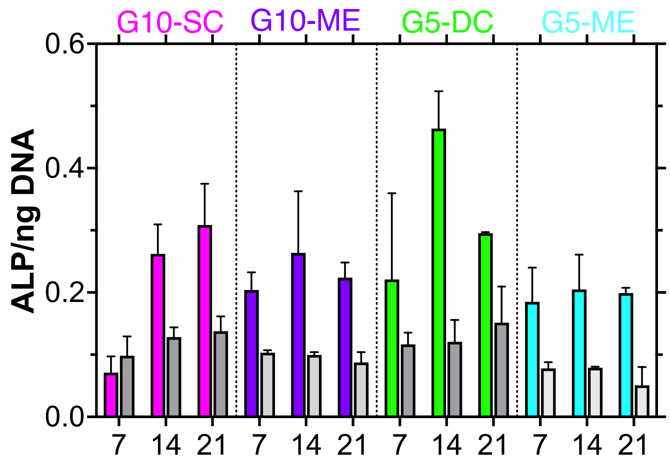

__Figure S6 Normalized ALP/DNA content for GelMA hydrogel samples:__ comparison of non-differentiated and differentiated sample.
